# A Rapid and Low-Cost PCR Thermal Cycler for Infectious Disease Diagnostics

**DOI:** 10.1371/journal.pone.0149150

**Published:** 2016-02-12

**Authors:** Kamfai Chan, Pui-Yan Wong, Peter Yu, Justin Hardick, Kah-Yat Wong, Scott A. Wilson, Tiffany Wu, Zoe Hui, Charlotte Gaydos, Season S. Wong

**Affiliations:** 1 AI Biosciences, Inc., College Station, Texas, United States of America; 2 Department of Medicine, Johns Hopkins University, Baltimore, Maryland, United States of America; Purdue University, UNITED STATES

## Abstract

The ability to make rapid diagnosis of infectious diseases broadly available in a portable, low-cost format would mark a great step forward in global health. Many molecular diagnostic assays are developed based on using thermal cyclers to carry out polymerase chain reaction (PCR) and reverse-transcription PCR for DNA and RNA amplification and detection, respectively. Unfortunately, most commercial thermal cyclers are expensive and need continuous electrical power supply, so they are not suitable for uses in low-resource settings. We have previously reported a low-cost and simple approach to amplify DNA using vacuum insulated stainless steel thermoses food cans, which we have named it thermos thermal cycler or TTC. Here, we describe the use of an improved set up to enable the detection of viral RNA targets by reverse-transcription PCR (RT-PCR), thus expanding the TTC’s ability to identify highly infectious, RNA virus-based diseases in low resource settings. The TTC was successful in demonstrating high-speed and sensitive detection of DNA or RNA targets of sexually transmitted diseases, HIV/AIDS, Ebola hemorrhagic fever, and dengue fever. Our innovative TTC costs less than $200 to build and has a capacity of at least eight tubes. In terms of speed, the TTC’s performance exceeded that of commercial thermal cyclers tested. When coupled with low-cost endpoint detection technologies such as nucleic acid lateral-flow assay or a cell-phone-based fluorescence detector, the TTC will increase the availability of on-site molecular diagnostics in low-resource settings.

## Introduction

The ability to make technologies for the rapid diagnosis of infectious disease broadly available in a portable, low-cost format would mark a revolutionary step forward in global public health [1, 2]. Access to decentralized molecular diagnostic testing can enable faster diagnostics, treatments, and subsequent control of infectious diseases. A critical challenge to efforts in decentralizing molecular diagnostic testing is that a large segment of the population in need of these advances resides in low-resource settings (LRS) that offer extremely limited laboratory infrastructure [3, 4]. While many well-characterized molecular assays have been developed around polymerase chain reaction (PCR) [5, 6], including pathogen and infectious disease detection [7–9], food and water safety [10–12], forensics [13, 14], population-scale polymorphisms [15, 16], and mutation studies [17], it remains largely a laboratory technique requiring expensive equipment and trained personnel. Thus, PCR-based devices for molecular diagnostics have not been appropriately commercialized for geographical areas and demographics that would benefit most from the technology. Besides the cost and temperature-sensitivity of reagents, thermal cyclers are generally much too expensive to be purchased for users in these areas. In addition, the timescales required to perform a typical PCR remain slow, generally about an hour or more. This is partially due to the large thermal masses of most commercial thermal cyclers that make the process very inefficient. To this end, many innovative methods aimed at performing PCR faster have been reported [18–36]. Although these approaches provide valuable scientific innovation, most of them are difficult to implement in LRS. In short, there remains an unmet need for a rapid and low-cost thermal cycler that can carry out molecular diagnostics in rural areas and developing countries in a rapid and low-cost manner.

**Fig 1:**
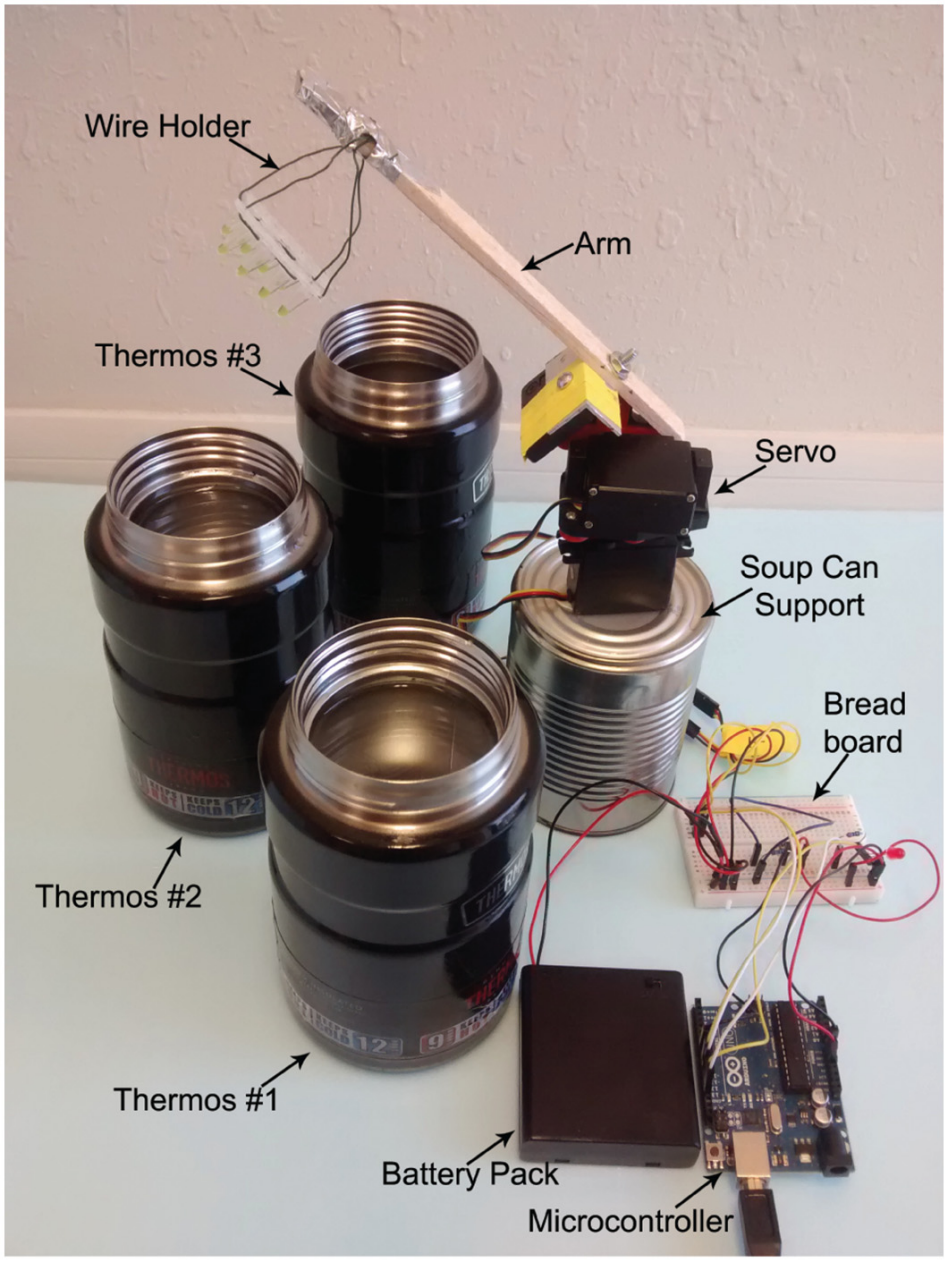
Setup of the low-cost and rapid TTC. Major components include three 24-oz thermoses and a pan-and-tilt servo set to control the up, down, and rotational motion to shuttle PCR vessels in and out of the thermoses. Also included are the battery pack, the Arduino electronic controller, and a breadboard. To reduce costs, the pan-and-tilt setup is constructed using a soup can for a fixed height, a wood stick, and a PCR tube holder made with metal wire.

We recently reported a practical solution for bringing a low-cost, simple-to-operate, and rapid thermal cycler to underserved and developing populations [37]. We demonstrated fast PCR thermal cycling at a rate of 15 to 30 s per cycle using a device that can be assembled for $130 using commercial off-the-shelf items [37]. This thermos thermal cycler (TTC) uses a very simple design that performs PCR amplification based on the “archaic” method of hand-transferring reaction tubes through a series of water baths, minimizing the temperature ramping time needed for PCR tubes to reach thermal equilibrium (Fig 1). The automation of the PCR reactions by an Arduino microcontroller is performed so that the PCR tubes are mechanically transferred by the actions of servomotors in order to carry out PCR steps. Our TTC does not need active thermal control, so it is highly suitable for use in LRS, where the continuous supply of electricity is often unreliable. Furthermore, our water-based TTC can accommodate all industry-standard PCR tubes, including 200 to 500 μL PCR tubes and plastic/glass capillary tubes (20 μL capacity) [38, 39]. In this paper, we extend the use of TTC to carry PCR and reverse-transcription PCR (RT-PCR) for the detection of infectious diseases. To carry out a RT-PCR for an RNA target, we added a thermos that is maintained between 40 to 50°C to perform reverse transcription. The use of an additional thermos increases the overall cost to slightly under $178.

## Materials and Methods

### Ethics Statement

De-identified clinical urine specimens were collected from patients who signed a written consent form from an earlier IRB approved study and who agreed to participate in the study and have their de-identified achieved sample be utilized in future research for the development of new molecular tests for STIs. Original written consent forms were stored in a binder in a locked filing cabinet with access provided to study team coordinator only. Both the consent process and study protocol were written in accordance with the approved guidelines set forth by Johns Hopkins University School of Medicine and Institutional Review Board protocols (IRB numbers: NA_00012998 and NA_00023037).

### Setting up water baths for TTC

For our typical reactions involving a two-step PCR, two 16- or 24-oz vacuum-insulated thermoses (thermos, catalog number NS340TL4 and SK3020MBTRI4, purchased from a local Target store) were used, with each thermos being prepared with water and oil at denaturation (95 to 97°C) or annealing/extension temperatures (60 or 65°C). A third thermos (50°C for reverse transcription) was also set up when RT-PCR was performed (Fig 1). The temperature of the water baths was measured using K-type thermocouples (Model No. SC-TT-K-30-36, Omega Engineering, Stamford, CT) connected to a data logger thermometer (HH147U, Omega Engineering, Stamford, CT). A portable immersion heater can be used to quickly reheat water in thermoses, allowing consecutive PCR runs. Detailed procedures on setting up the thermoses to the desired temperatures and automating the TTC for PCR runs can be found in S1 File and our earlier publication [37].

### Speeding up PCR reactions when polypropylene tubes are used

While polypropylene plastic tubes are commonly used in most commercial thermal cycles, they are not ideal for achieving a fast PCR reaction time because of the slow heat transfer between the plastic and the heating element. This is especially true when the tubes are cooled from denaturation temperature to annealing/extension temperature; we have found that most thermal cycles need 10 to 12 s to switch between 95 and 60°C. To reduce the time needed to cool the reagent for annealing in the TTC setup, we added a room-temperature bath (RTB) between the denaturation and annealing/extension bath during down-ramping. The RTB rapidly lowered the temperature of the reagents inside the PCR tubes from over 90°C to just above 60°C in 2.5 s before lowering the tubes into the 60°C bath (Figure C in S1 File).

### General PCR conditions

All commercial thermal-block-based PCR runs were performed in a CFX96 Touch real-time thermal cycler (Bio-Rad) or SimpliAmp thermal cycler (Life Technologies). Polypropylene plastic tubes and glass capillary tubes were also used to demonstrate the TTC’s flexibility in terms of accepting various tubes to deliver fast heat transfer in water. While there is no heated lid in our design, and we noticed that some evaporation and condensation occurred, we found that the quality of the PCR amplification was not significantly affected when checked by gel electrophoresis. In this manuscript, the template concentrations used in PCR reactions were purposely chosen to be very realistic, with most of them giving a Cq of 25 to 34, which allows us to access and demonstrate the efficiency of the TTC. Table 1 summarizes the targets, primer sequences, product sizes, and components of the PCR reactions. The individual PCR and RT-PCR run protocols will be described in the corresponding data sections. Stock primer concentrations were 10 μM and were diluted to a final concentration of 500 nM after the master mixes were prepared. Unless specified otherwise, reaction volumes for the commercial cycler and TTC were 20 μL when polypropylene tubes were used. The sample volume for glass capillary tubes was 17 μL.

**Table 1:** Target, primer sequences, target sizes, and components of PCR.

Target (PCR amplicon sizes)	PCR components
**Human KCNE1 (45 bp)**	10 μL 2x PrimeSTAR Max mix or iTaq Universal SYBR PCR mix
	1 μL For primer (5’ CCC ATT CAA CGT CTA CAT CGA GTC 3’)
	1 μL Rev primer (5’ TCC TTC TCT TGC CAG GCA T 3’)
	2 μL SpeedSTAR HS polymerase
	4 μL nuclease-free water
	2 μL template
	Total volume 20 μL (use 10 μL per reaction)
**Human DNA (600 bp, 500 bp, 400 bp, 300 bp, 200 bp, 100 bp)**	7.5 μL Isohelix DNA Quality Check Kit human primer mix
	12.5 μL Isohelix DNA Quality Check Kit amplification mix
	2.5 μL nuclease-free water
	2.5 μL human genomic DNA
	Total volume 25 μL
***Chlamydia trachomatis* 16S rRNA gene (165 bp)**	10 μL 2x Premix Ex Taq
	1 μL For primer (5’ TAG GCG GAT TGA GAG ATT GG 3’)
	1 μL Rev primer (5’ TAT TCC CAA GCG AAA GTG CT 3’)
	0.5 μL Probe (5’ AGA ATC TTT CGC AAT GGA CG 3’)
	5.5 μL nuclease-free water
	2 μL template
	Total volume 20 μL
**HIV gag (~85 bp)**	10 μL 2x One Step RT-PCR buffer III
	0.5 μL PrimeScript RT enzyme mix II
	0.5 μLTakara Ex Taq HS
	1 μL For primer (5’ GGC TAC ACT AGA AGA AAT GAT GAC AGC AT 3’)
	1 μL Rev primer (5’ GCT CAT TGC TTC AGC CAA AAC TCT TGC 3’)
	0.5 μL Probe (5’ AGT AGG AGG ACC CGG CCA TA 3’)
	2.5 μL nuclease-free water
	4 μL template (at various concentrations)
	Total volume 20 μL
***Ebola virus* NP (112 bp)**	10 μL 2x One Step RT-PCR buffer III
	0.5 μL PrimeScript RT enzyme mix II
	0.5 μLTakara Ex Taq HS
	1 μL For primer (5’ AGG GTG ATC CAA CAA CCT TAA T 3’)
	1 μL Rev primer (5’ CTC TGG GTG TTC AGG TTC AAA 3’)
	0.5 μL Probe (5’ TGC TTG TTT GAC TGT GAA CTA ATG CTG TC 3’)
	2.5 μL nuclease-free water
	4 μL template
	Total volume 20 μL
***Dengue virus* I (112 bp)**	12.5 μL 2x One Step RT-PCR buffer III
	0.5 μL PrimeScript RT enzyme mix II
	0.5 μLTakara Ex Taq HS
	0.5 μL For primer (5’ CAA AAG GAA GTC GYG CAA TA 3’)
	0.5 μL Rev primer (5’ CTG AGT GAA TTC TCT CTG CTR AAC 3’)
	0.45 μL Probe (5’ CAT GTG GYT GGG AGC RCG C 3’)
	8.05 μL nuclease-free water
	2 μL template
	Total volume 25 μL

### Gel electrophoresis analysis

After amplification by the TTC or the commercial unit, the PCR amplicons were typically evaluated using a 2.2% pre-casted gel (Flash Gel by Lonza) at 275 V for 7 min (4 μL mix added to 1 μL of loading dye). The resulting gel images were captured using a cell phone camera. The typical ladder sizes used in the gel were 50, 100, 150, 200, 300, 500, 800, and 1500 bp.

### Demonstrating the speed of reaction by the TTC

To demonstrate the speed of the TTC system, a 45-bp fragment of the single-copy gene KCNE1 (potassium channel, voltage-gated Isk-related subfamily E regulatory beta subunit 1) was amplified from human genomic DNA by the TTC using primers reported in another study [25]. We note that this is a very small fragment, so we expect that PCR can be completed relatively fast. Using the TTC with glass capillary tubes, we empirically reduced the time spent in the thermos with a 30-s hot-start, followed by 40 cycles of 97°C (8 s) and 60°C (10 s). The shortest time we tried that obtained PCR amplicons was 2-s denaturation and 4-s annealing/extension (~10 cycles per min, or 4 min for 40 PCR cycles). After the reactions performed with SYBR Premix Ex Taq (Takara/CloneTech), the tubes were checked by viewing with a blue LED gel illumination box (Lonza), with the fluorescent signal captured by an orange filter placed in front of a cell phone camera.

### Multiplexed PCR reactions

To demonstrate the TTC’s ability to carry out multiplexed reactions (6-plex) and multiple reactions (eight) in a single run, a commercially available multiplexed PCR kit (Isohelix DQC Kit) was purchased, and the positive control human DNA template was used in PCR reactions. The Isohelix kit typically yields six proprietary amplicons at 100, 200, 300, 400, 500, and 600 bp. The 500-bp fragment is derived from an internal control and is present even in no-template controls. Commercial PCR reactions were performed in the following conditions: 5 min of hot-start at 95°C, followed by 35 cycles of 95°C (30 s), 65°C (30 s), and 72°C (45 s). The total run time was 84 min. Eight TTC-based reactions were performed simultaneously using three water baths. The first water bath was for denaturation, while the second water bath was maintained at room temperature to help quickly cool the reaction tubes close to 65°C. The tubes were then transported to the third bath, maintained at 65°C. Unlike the manufacturer’s suggested protocols, we opted to combine the annealing and extension step at 65°C. We shortened the incubation time in each water bath: a 3-min hot-start at 97°C, followed by 35 cycles of 95°C (15 s), 25°C (2.5 s), and 65°C (20 s). We referred to this protocol as 180s/35x(15s/2.5s/20s), which took the TTC 28 min to complete.

### Demonstrating PCR amplification using urine samples

To demonstrate the practical value of the TTC, we used it to amplify genomic bacterial DNA extracted from clinical samples of previously collected urine samples found to be positive with CT. Serially diluted CT positive urine samples 1X, 1/10, 1/100, and 1/1000 were extracted by our in-house-developed, magnetic-particle-based extraction protocol. The eluted DNA templates were prepared in Premix Ex Taq (Probe qPCR) polymerase master mix (Takara/Clontech) and amplified in glass capillary tubes with a TaqMan hydrolysis probe assay. Commercial PCR reactions were performed with the following conditions: 30 s of hot-start at 95°C, followed by 40 cycles of 95°C (5 s) and 60°C (15 s). This took 43 min to complete 40 cycles. The TTC-PCR was performed with the following conditions: a 30-s hot-start at 97°C, followed by 35 or 40 cycles of 95°C (5 s) and 60°C (10 s). The PCR amplicon (165 bp) was checked with gel electrophoresis. The progress of the real-time PCR using TTC can also be monitored by using blue LEDs for dye excitation and digital or cellphone camera with orange filter to take photos of the glass capillary tubes after each cycle. The green fluorescence from samples with higher template concentrations should rise up faster than the ones with less templates.

### Demonstrating detection of HIV by RT-PCR with the TTC

To demonstrate that our TTC is robust and versatile, we performed RT-PCR with extracted HIV RNA. Cultured HIV-1 Type B virus (8E5) in HIV-1 RNA negative, defibrinated human plasma was used as our sample (ACCURUN 315 Series 500, SeraCare). The 8E5 virus contains an intact but defective viral genome. This control is formulated for use with in-vitro diagnostic test methods that detect and quantitate HIV-1 RNA. The 500 series contains 130,000–300,000 copies/mL of sample. RNA extraction was carried out by a NucliSENS protocol or by using a spin-column extraction method by Qiagen. The extracted template was tested by qRT-PCR using a SuperScript III Platinum One-Step Quantitative RT-PCR system (Life Technologies) or a One Step PrimeScript RT-PCR Kit (Takara/Clontech). A commercial thermal cycler was used to perform real-time RT-PCR, with a 5-min reverse-transcription step followed by 30 s of reverse transcriptase inhibition and polymerase actuation at 95°C. The cDNA was amplified with 45 cycles of 95°C (5 s) and 60°C (15 s). This 300s/30s/45x(5s/15s) reaction took ~45 min to complete. Primers and probe information are listed in Table 1. With the TTC, we performed numerous reactions in both propylene PCR tubes and glass capillary tubes. For reactions carried out in thin-walled polypropylene PCR tubes (Cat. No. 16950, Sorenson Bioscience), we used the following typical protocol: 300s/10s/45x or 50x (10s/30s) of RT process, followed by 10 s of RT inhibition, and then 45 or 50 cycles of 10 s of denaturation and 30 s of annealing/extension. The shortest protocol we tried and that gave a high performance of target amplification was 300s/30s/45x(5s/10s). We used 45 instead of 40 cycles in performing RT-PCR because we wanted to show that the TTC can handle very low template copies (<10 copies per reaction) since the threshold cycles of commercial PCR run were over 30 (32 to 36 in most cases).

When RT-PCR was performed, an additional thermos was needed for the reverse-transcription step. A TTC with pan-and-tilt servos was used to rapidly and precisely shuttle the reaction tubes between the RT bath, RT inhibition/cDNA denaturation bath, RTB bath, and annealing/extension bath (four containers total).

### Demonstrating detection of recombinant Ebola virus RNA

Outbreaks of new viral communicable diseases and emergence of new viral strains, such as the recent outbreak of the Ebola 2014 strain in Guinea, can represent public health emergencies. Rapid and sensitive PCR assays can help diagnose and treat the infected while helping to contain a rapid outbreak. Therefore, we used a positive reference material to demonstrate our ability to amplify RNA in low-resource settings using the TTC. RNA from recombinant Ebola virus (AccuPlex rEbola GP/NP, SeraCare) was obtained for RT-PCR by the TTC after an in-house 15-min extraction was processed. The synthetic constructs were designed targeting the glycoprotein gene (GP), nucleoprotein gene (NP), and VP24 of Ebola Zaire isolate (H.sapiens-wt/GIN/2014/Gueckedou-C05; GenBank accession number KJ660348.2). The capped RNA was introduced into baby hamster kidney cells. The recombinant virus was cultured and purified, heat-inactivated, and then diluted into defibrinated human plasma and 0.09% sodium azide as a preservative. Primers and probe information are listed in Table 1.

The extracted RNA was tested using a lab-developed research-use-only assay developed by PrimerDesign (Southampton, United Kingdom) and SeraCare (Milford, MA). The TTC RT-PCR was performed using protocols similar to the HIV test, with PCR tubes transferred between three thermoses (reverse transcription, denaturation, and annealing/extension) and an optional room-temperature water bath. The TTC-based PCR conditions involved 5 min of RT processing followed by 10 s of RT enzyme inhibition and cDNA denaturation. Forty-five to 50 cycles of PCR reactions (with various incubation times) were then performed.

### Demonstrating rapid amplification of dengue virus RNA

The CDC has recently developed a new diagnostic test, “CDC DENV-1-4 Real Time RT PCR Assay,” for the detection and serotype identification of dengue [40]. This is the first FDA-approved molecular test for dengue that detects evidence of the virus itself. The test can identify all four dengue virus types [40, 41]. This new test will help diagnose dengue within the first 7 days after symptoms of the illness appear; which is the period when people are most likely to see a health care professional. We used the positive samples in this kit, which include heat-inactivated dengue virus RNA in human serum, to extract RNA for RT-PCR reactions. The extracted total RNA samples were amplified by a commercial cycler and the TTC. The commercial RT-PCR condition is listed in Table 1.

## Results

### Demonstration of TTC’s speed

**Fig 2:**
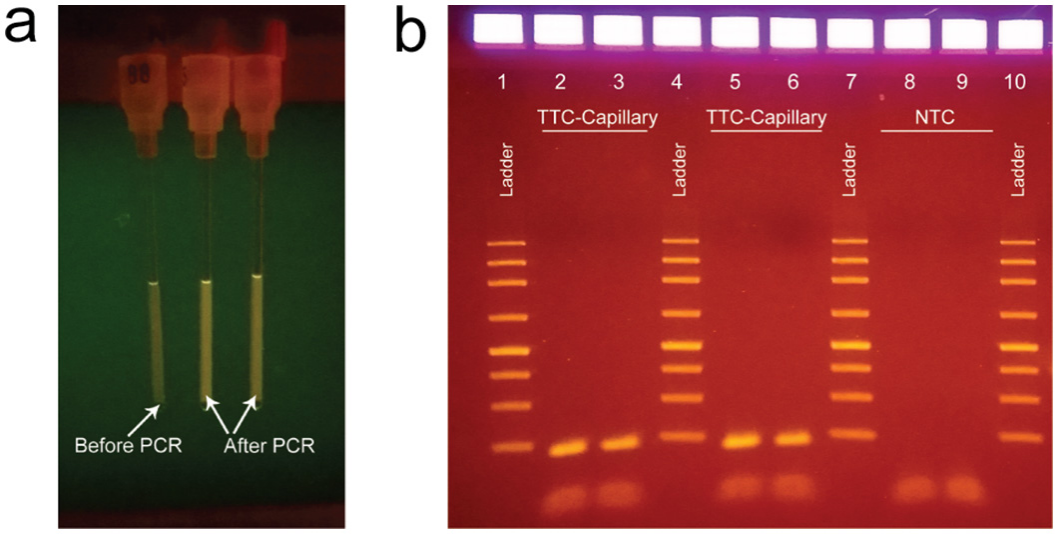
**(a) Fluorescent intensity of capillary tubes before after PCR**. The two tubes on the right are capillary tubes after TTC-PCR [30s/40x(4s/6s) in under 7.5 min]. **(b) Gel electrophoresis data after rapid TTC-PCR**. Lane 1: ladder. Lanes 2 and 3: duplicate samples that had PCR cycles of 30s/40x(4s/6s) that took under 7.5 min to complete. Lane 4: ladder. Lanes 5 and 6: duplicate samples that had PCR cycles of 30s/40x(2s/4s) that took 5 min to complete. Lane 7: ladder. Lanes 8 and 9: NTC. Lane 10: ladder.

A 45-bp fragment of the single-copy gene KCNE1 was amplified from human genomic DNA by the TTC. With a normal concentration of primers (500 nM), 40 cycles of rapid PCR can generate PCR amplicons for gel identification using the 30s/40x(2s/4s) (under 5.5 min) protocol, although the result is slightly more consistent with 30s/40x(4s/6s) (under 7.5 min). In contrast, commercial PCR needs 40 min for completion. Fig 2a is an image of the glass capillary tubes after 30s/40x(4s/6s) PCR reactions (total run time of 7.5 min). The capillary tube on the left did not undergo PCR, while the two tubes on the right show reactions after TTC-PCR. It is clear that the fluorescent signal at the conclusion of PCR was higher due to the hydrolysis of the probes. The gel electrophoresis shown in Fig 2b confirms that the correct amplicons were produced with both the 5.5- and 7.5-min protocols using glass capillary tubes. These results confirm that the performance of our TTC was very impressive in terms of the cost-to-build and the speed with which reaction can be accomplished. Based on a recent report by the Wittwer group, we expect extremely fast PCR (15 to 60 s per run) can be achieved using the TTC if 20-fold more concentrated primers and enzymes are used [25].

### Multiplex PCR amplification using TTC

**Fig 3:**
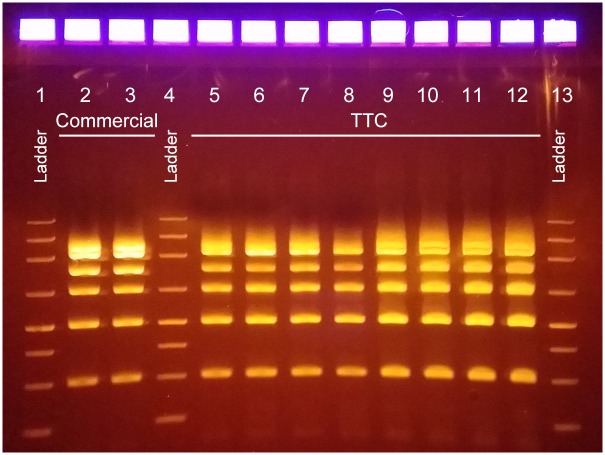
Eight identical multiplexed PCR reactions performed with TTC. Lane 1: ladder. Lanes 2 and 3: amplicons produced by the commercial thermal cycler (run time of 84 min). Lane 4: ladder. Lanes 5 to 12: amplicons produced by the TTC using a 180s/35x(15s/2.5s/20s) protocol (run time of 28 min). Lane 13: ladder.

Using the Isohelix DNA Quality Check (DQC) Kit, we ran the PCR using TTC and used gel electrophoresis to confirm that multiple targets could be amplified. The gel photo in Fig 3 shows that the TTC can produce multiplexed amplicons with the correct sizes and that the yield is similar to a three-step reaction performed in the commercial cycler with same number of PCR cycles. In addition, all eight samples that were run simultaneously produced very good amounts of products, indicating that the TTC can accept up to eight samples and provide homogenous conditions within the thermos for PCR. The 28-min reaction time needed by the TTC to perform 35 cycles is much shorter than the protocol performed with a commercial thermal cycler using the manufacturer’s suggestions (85 min for 35 cycles).

### PCR amplification using clinical samples

**Fig 4:**
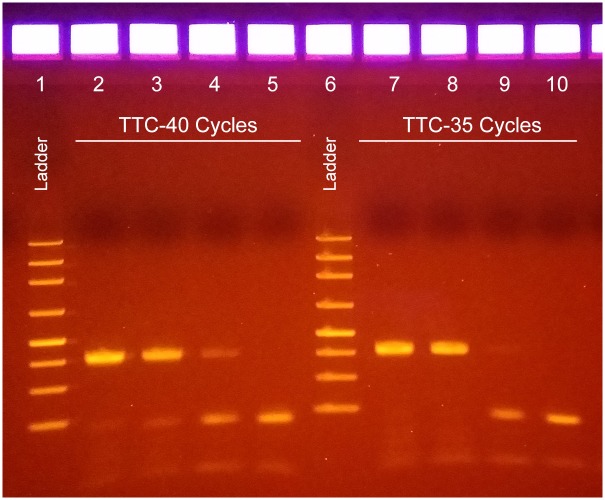
PCR amplification of *Chlamydia trachomatis* DNA template from clinical samples. Lane 1: ladder. Lanes 2 to 5: templates from 1 to 1000X diluted samples amplified for 40 cycles (under 12 min to complete the PCR). Lane 6: ladder. Lanes 7 to 10 contain the same samples as Lanes 2–5 but were only amplified for 35 PCR cycles (10.5 min).

To demonstrate TTC’s practical value, we used it to amplify genomic DNA extracted from *Chlamydia trachomatis* (CT) positive urine samples. We extracted serially diluted CT) from positive urine samples at 1, 1/10, 1/100, and 1/1,000 of the original concentration using a protocol developed in house, and the template was then amplified in glass capillary tubes with a TaqMan hydrolysis probe assay. The TTC-PCR reactions that produce 132-bp amplicons were performed with the following condition: 30-s hot-start at 97°C, followed by 35 or 40 cycles at 95°C (5 s) and 60°C (10 s). The amplicons were checked by gel electrophoresis (Fig 4). Photos of the glass capillary tubes after each cycle can be recorded to monitor the progress of increasing green fluorescence in tubes with positive target template. An example PCR reaction was recorded and photos were compiled into a gif file as supporting information (S1 Video). TTC was able to amplify clinical, sample-derived bacterial DNA in a <12-min (40-cycle) protocol, even though a 10.5-min (35-cycle) protocol already can produce enough amplicons to be visible by gel electrophoresis imaging. In contrast, commercial PCR reactions took 45 min to complete a 40-cycle reaction (not shown).

### RNA amplification for Ebola detection using TTC

While we have successfully demonstrated the use of TTC for DNA, the amplification of RNA is also critical and, in general, more complicated for point-of-care or low-resource settings since it needs an additional reverse-transcription process prior to PCR. Also, many infectious disease diagnostics rely on the detection of viral RNA (e.g., HIV, Ebola, dengue viruses), so it is critical that the TTC can handle RNA templates by performing one-step RT-PCR, especially for emerging RNA viruses (such as Middle East Respiratory Syndrome coronavirus-specific RNA) [42, 43].

Using our TTC, which is equipped with a pan-and-tilt servo kit, users can move PCR tubes into the ≥3 thermoses/containers needed to optimally run this reaction. For traditional one-step RT-PCR, four water baths for reverse transcription, denaturation, annealing, and extension can be prepared. Alternatively, annealing and extension steps can be combined, so users can perform reverse transcription in one thermos, followed by denaturation in another thermos, and then use a third thermos for annealing/extension. When polypropylene tubes are used, we can perform reverse transcription in a thermos, then perform denaturation in another thermos, then use a third room-temperature water bath (a thermos is not necessary) to quickly drop the temperature from over 90°C to slightly above annealing/extension temperature, and finally use a fourth thermos for annealing/extension. This helps speed up the reactions due to faster heat transfer.

**Fig 5:**
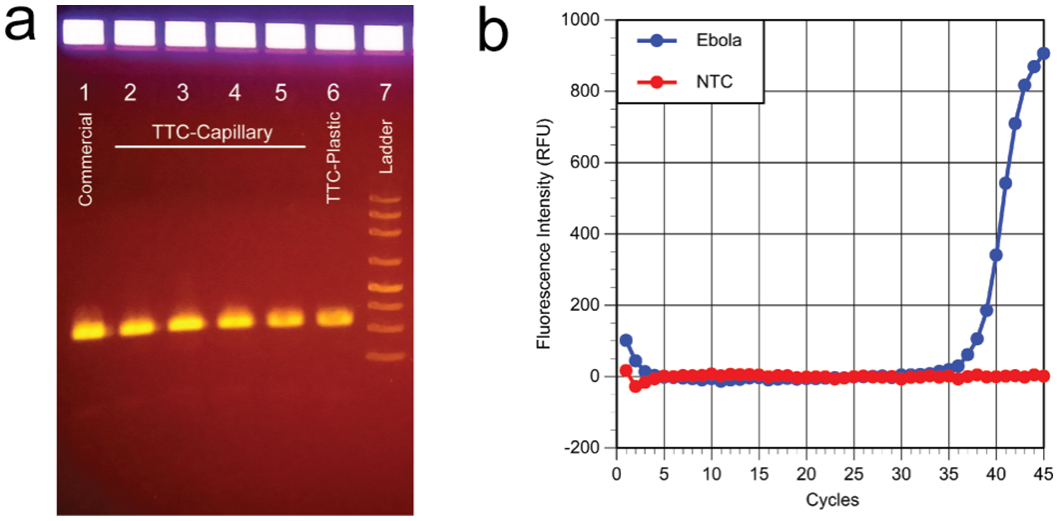
**(a) Gel data for Ebola RNA after RT-PCR**. Lane 1: commercial PCR 300s/30s/45x(30s/10s/30s), run time of 69 min. Lanes 2 and 3: TTC run with glass capillary tubes 300s/10s/50x(9s/21s), total run time of 31.9 min. Lanes 4 and 5: TTC run with glass capillary tubes 300s/10s/45x(12s/25s), total run time of 34.5 min. Lane 6: TTC run with plastic tubes 300s/10s/45x(20s/30s), total run time of 44.2 min. Lane 7: ladder. The gel data show that the amplification produced the correct product, and the yield is similar to commercial PCR runs. **(b) Real-time RT-PCR plot from the commercial run**. The Cq of 36.8 suggests that the TTC can amplify very small amounts of RNA.

Using a three-bath system and before optimizing the run protocol for speed, we were able to amplify and detect recombinant Ebola RNA in glass capillary tubes in under 32 min. The gel picture in Fig 5a shows that the yield from rapid RT-PCR is about the same as commercial products. However, the RT-PCR reactions in plastic tubes can be completed (including a 5-min reverse-transcription process) within 44.2 min vs. 69 min in the commercial unit. Our results support the notion that high-quality, low-resource setting molecular diagnostics can be achieved with a low-cost device.

It is important to note that the Cq of the PCR reaction demonstrated by the real-time RT-PCR plot was over 35 cycles, meaning that the number of targets in each PCR reaction is likely below 10 copies (Fig 5b). The ability of the TTC to produce enough amplicons for gel electrophoresis means that the efficiency of the TTC is quite high. While others have reported fast PCR reactions using various approaches, seldom were the systems challenged with PCR reactions containing low numbers of template per PCR reaction. This supports our belief that the efficiency of PCR performed by the TTC is very close to that of a commercial unit. The ability of this low-cost TTC to perform highly sensitive RT-PCR amplification of <10 template copies of RNA is quite remarkable.

### HIV RNA amplification

**Fig 6:**
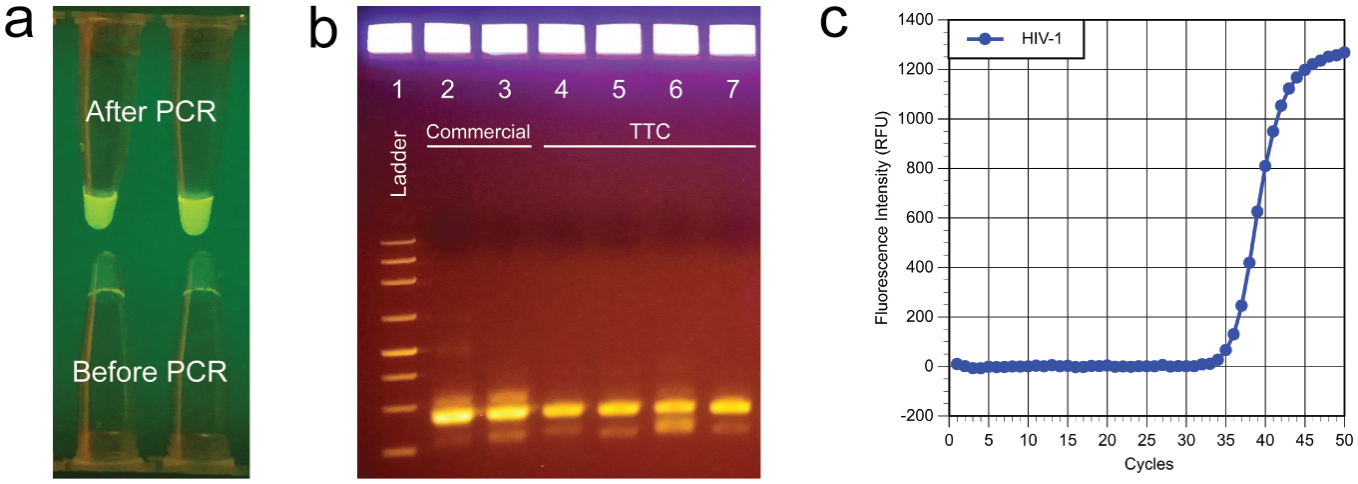
**(a) Fluorescent signal from reactions using plastic tubes**. The PCR tubes that had undergone 45 cycles of TTC RT-PCR amplification were visualized using a cell phone camera. The top two tubes are HIV positive samples, while the bottom two tubes are the same mix that did not go through the RT-PCR process. **(b) Gel electrophoresis data of HIV RNA amplicons after RT-PCR using thin-walled polypropylene tubes**. Lane 1: ladder. Lanes 2 and 3: commercial RT-PCR (50 cycles in 83 min). Lanes 4 and 5: TTC 330s/10s/45x(15s/30s) (41 min). Lanes 6 and 7: TTC 330s/20s/45x(15s/30s) (41.3 min). **(c) Real-time PCR data from the commercial thermal cycler (Cq = 34.8)**.

**Fig 7:**
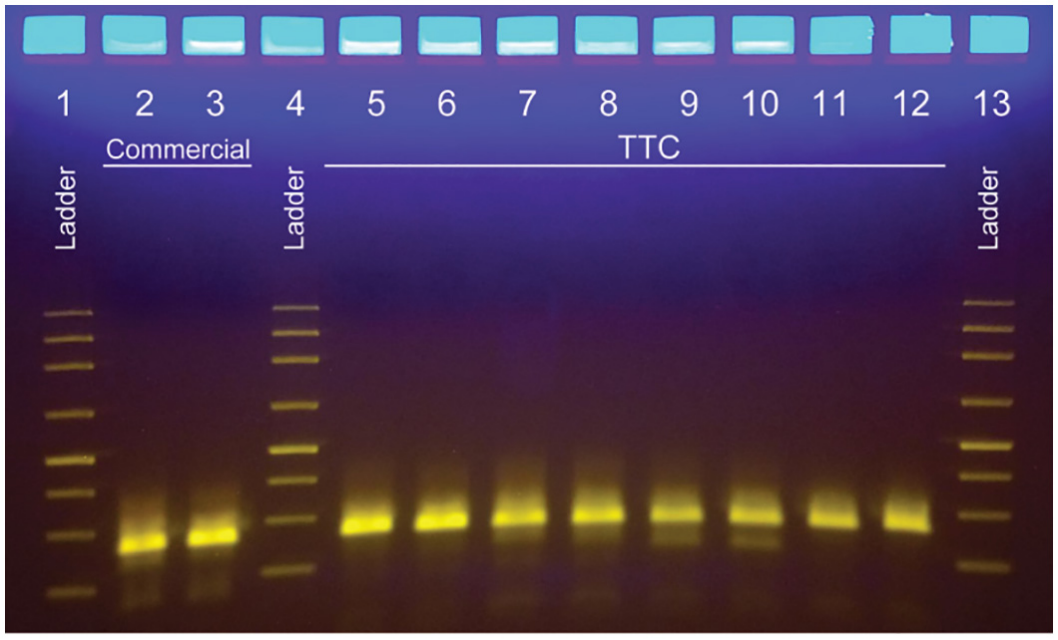
Gel electrophoresis data of HIV RNA amplification in glass capillary tubes. Lane 1: ladder. Lanes 2 and 3: commercial run at 300s/30s/45x(10s/30s) in 74 min. Lane 4: ladder. Lanes 5 and 6: 300s/10s/45x(9s/21s) in 28.5 min. Lanes 7 and 8: 300s/10s/40x(9s/16s) in 24.8 min. Lanes 9 and 10: 300s/10s/45x(9s/11s) in 22.1 min. Lanes 11 and 12: 300s/10s/45x(5s/10s) in 17.3 min. Lane 13: ladder. Although the amount of amplicons generated was reduced when shorter protocols were used, a reasonable amount of amplicons was still produced.

We also used the TTC for HIV virus detection by amplifying RNA extracted from HIV-1 in serum. The expected product size of the reaction is 85 bp. The commercial PCR thermal cyclers showed that RT-PCR can be completed in about 84 min with a Cq of 35 in a 50-cycle reaction. Fig 6a shows that RT-PCR of HIV RNA can be amplified in polypropylene PCR tubes using the TTC. The two upper tubes contain target RNA, while the bottom two tubes are samples that did not go through the reactions. This reaction was performed using the following protocol: 420s/10s/45x(15s/2.5s/30s). During the TTC RT-PCR run (46 min), the denaturation bath dropped from 97.4°C to 92.7°C, while the annealing/extension bath dropped from 61.6°C to 59.2°C. The temperature drops were not severe enough that they affected PCR results severely. Fig 6b is the representative gel data showing the amplicons after PCR reactions in propylene tubes. The first two samples in the gel picture are results of commercial RT-PCR using a 50-cycle protocol (total run time of 84 min). The next two samples (Lanes 4 and 5) are amplicons from a 45-cycle TTC RT-PCR run using a protocol of 330s/10s/45x(15s/30s) and a total run time of 41 min. In another run, TTC was able to complete 45 cycles of RT-PCR in 41.3 min using a protocol of 330s/20s/45x(15s/30s). During the run, the temperature of the denaturation thermos dropped 4.2°C, while the annealing/extension thermos dropped 1.5°C. Therefore, TTC can produce the correct-sized amplicons in about half the reaction time of a commercial unit. We note that because the commercial run included five more cycles than the TTC runs, it is expected that the gel bands’ intensities are higher than those from the TTC. Fig 6c is the real-time PCR plot of the commercial run (50 cycles). Next, glass capillary tubes were used to perform RT-PCR for HIV detection. Fig 7 summarizes the results with a range of reaction conditions. In these runs, the RT period (300 s) and the RT inactivation period (10 s) were identical, and only the annealing and extension time were modified. Using a protocol of 9 s denaturation and 21 s annealing/extension, TTC completed 45-cycle runs (protocol at 300s/10s/45x[9s/21s]) in 27.7 min. Even when the protocol was reduced to 300s/10s/40x(5s/10s) with a total run time of 16.4 min for a 45-cycle RT-PCR reaction (Lane 10 and 11), amplicons were still being produced, even though the overall efficiency seems to have dropped slightly. Overall, the gel data demonstrate that the TTC was able to complete 45 cycles of RT-PCR using the three-bath TTC targeting sub-100 bp HIV amplicons. As a reference, a 45-cycle reaction performed by the commercial cycler took 74 min.

### Dengue virus RNA amplification

Next, we used the TTC to demonstrate possible dengue diagnostics by RT-PCR using the RNA extracted from inactivated dengue in human serum. Similar to the amplification of RNA from HIV and recombinant Ebola virus, the TTC can carry out effective dengue RNA amplification in both plastic and glass capillary PCR tubes. The product size range of the dengue amplicons (I to IV) in the CDC multiplex RT-PCR reactions is between 74 to 112 bp, but only primer and probe targeting DENV-1 was used in this study. The gel electrophoresis data (not shown) demonstrated that TTC can be effectively performed with the following protocol: 300s/10s/45x(8s/16s) (total run time of 25 min) in glass capillary tubes and 300s/15s/40x(12s/18s) (total time of 26.3 min) in plastic tubes. The commercial RT-PCR used a protocol of 300s/10s/45x(8s/16s), which took 53 min to complete and had a Cq of 33.4.

**Fig 8:**
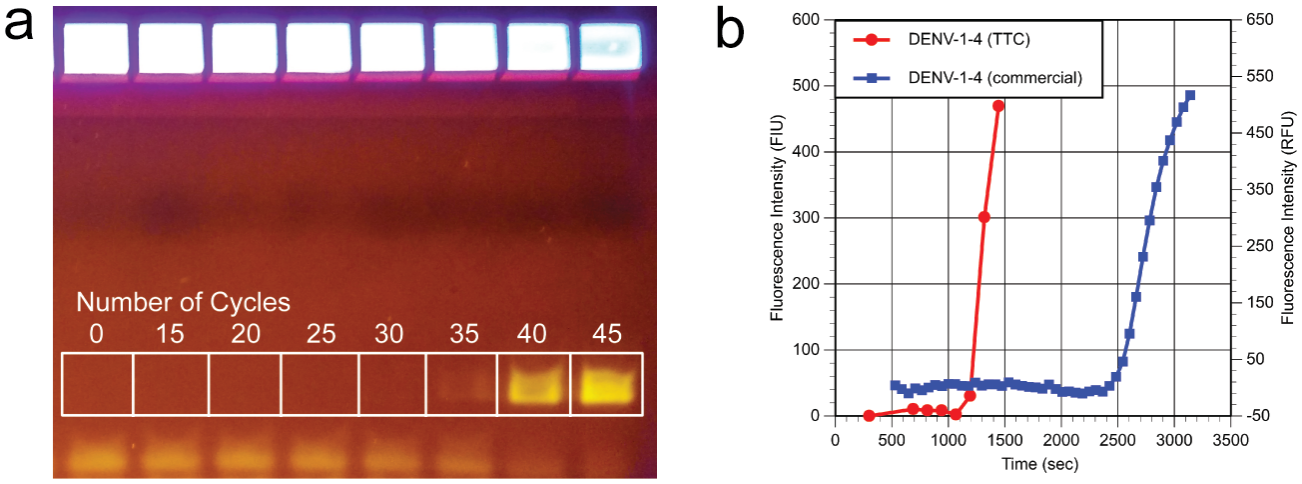
**(a) Gel electrophoresis data of the RT-PCR amplification of DENV-1 RNA at different number of PCR cycles**. The gel data show that the signal grew exponentially starting between cycles 30 to 35. **(b) Real-time plot of TTC vs. commercial cycler using the same PCR mix from (a)**. The time to complete the specific cycle was plotted against the fluorescence of the PCR mix post-PCR. It took 1068 and 2424 s for TTC and commercial thermal cycler to reach the Cq level, respectively.

Besides the gel electrophoresis of amplicon post-PCR, we monitored the progress of amplification by running multiple PCRs that each received a different number of cycles after the same reverse-transcription process. We chose a low template concentration (Cq of 33.4 from a commercial run) to ensure that the PCR did not reach the plateau phase too early and mask the real-time intensity difference between runs. Fig 8a is the gel electrophoresis data of a series of 300s/10s/45x(8s/16s) reactions showing the amplicons at cycle 0, 15, 20, 25, 30, 35, 40, and 45. It is clear that the amplicon gel band did not show up until the cycler number reached 35; therefore, the Cq is likely between 30 and 35. Next, we converted the cycler number information to a time-scale in order to illustrate the speed of the TTC (Fig 8b). The fluorescence signal from the TTC (measured by a portable fluorescent detector [Tubescanner, Qiagen]) and the commercial run was plotted against the reaction time. It is very clear that the TTC signal rose above the background signal much faster (1068 s, or 17.8 min, after RT started) than the commercial run (2424 s, or 40.4 min), even though the Cq values were similar. The results clearly indicated that the TTC is a much faster thermal cycler for generating a positive signal in the real-time reactions.

### Further improvement and energy consumption of the TTC

While PCR performed by the TTC has been conducted without using any enclosure, it is expected that in order for it to perform well in low-resource settings with extreme temperature range, an enclosure should be added. We note that, while our setup is more than enough to perform PCR rapidly and with great results, we can further stabilize the temperature by simply adding a Styrofoam box to enclose the TTC, placing a small metal can inside, and filling it with boiling water. This adds little cost and complexity to the TTC runs. The heat released by the near-boiling water quickly warms the air mass inside of the box. With the increased air temperature, the thermal gradient between the water and the air becomes smaller, which can lead to a reduced temperature drop in both thermoses.

We also tested the TTC’s water baths’ ability to maintain temperature by placing them inside a refrigerator (8°C) and in the cargo space of an SUV under a hot sun (47°C) for 1 hr. When placed inside a refrigerator, the temperature of the denaturation and annealing/extension baths dropped 5.8 and 2.4°C in 60 min. When placed inside a hot SUV, the temperature of the denaturation and annealing/extension baths dropped only 3.5 and 1.0°C in 60 min. The smaller temperature drop in a hot environment was expected due to the smaller thermal gradient between the water and the environment.

While energy input (electricity, propane gas, or even wood fire) is needed to heat water for the first run, subsequent reheating of water uses very little energy since the temperature in each thermos only drops a few degrees by the end of each run. We have calculated that 10 normal PCR runs in the TTC would use only 0.44 kWh worth of electricity. We also have calculated that the total cost of electricity for 10 consecutive runs can be as low as 5.3 or 8 cents in the United States or Nigeria, respectively. In contrast, at least 1.6 kWh will be consumed if PCR runs are performed using a regular thermal cycler (e.g., the SimpliAmp from Life Technologies).

## Discussion

The combined use of mineral oil and multiple vacuum-insulated, stainless steel thermoses in our TTC enables the capability to maintain water temperatures stable enough to perform rapid PCR and RT-PCR without any active temperature control during the reactions. This simple platform can be used in resource-limiting settings where stable or continuous power supply is not guaranteed. Specifically, we demonstrated that our innovative TTC can potentially deliver high-speed and sensitive PCR and RT-PCR for the amplification and detection of infectious diseases such as sexually transmitted diseases (chlamydia/gonorrhea), HIV/AIDS, Ebola hemorrhagic fever, and dengue fever. We also demonstrated that the TTC easily accommodates eight samples simultaneously, and we successfully performed highly multiplexed reactions. By designing the reaction to produce short strands of amplicons (45 bp), we also demonstrated that a 6-s per cycle PCR is feasible without using high concentrations of PCR reagents to speed up the reactions, thus making it one of the fastest and most economical thermal cycling units reported in the literature. In addition, we demonstrated that, when using propylene tubes, the duration of PCR runs can be further shortened by employing an extra room-temperature water bath to rapidly cool the PCR reagents from the denaturation temperature to near the annealing temperature. This simple step can cut 10 s off each PCR cycle without compromising PCR efficiency. Since the volume and PCR tubes we used are typical to lab-based PCR, little assay optimization is needed to run PCR using the TTC. With the advantages we have demonstrated, the TTC system could increase the availability of on-site molecular diagnostics in low-resource settings when coupled with low-cost, endpoint-detection technologies such as nucleic acid lateral-flow assay or a smartphone-based fluorescence detector. We are working toward equipping the TTC with real-time capability by adding a fluorescence detection system using LEDs for fluorescent excitation and using a smartphone for fluorescent signal monitoring.

## Supporting Information

S1 File Detail description on how to construct and program the TTC, Arduino codes for the 4-bath setup to run RT-PCR with room temperature bath, and room-temperature bath can speed up PCR when plastic tubes are used. (DOCX)

S1 Video This is a gif file showing the progress of real-time PCR in glass capillary tubes. The left tube has the highest template concentration (1X), the second tube has 1/10 diluted template, the third tube has 1/1000 diluted template and last tube on the right is the no-template-control sample. As expected, it took the least number of cycles for fluorescence signal in the left tube to rise above the background signal. The fluorescence in no-template control tube remained low throughout the reaction.
(GIF)
