# Supplementary material for: A Rapid and Low-Cost PCR Thermal Cycler for Infectious Disease Diagnostics
Source: PLoS One. 2016 Feb 12;11(2):e0149150. doi: 10.1371/journal.pone.0149150 (PMC4752298; doi:10.1371/journal.pone.0149150)
Supplement: S1 File — (DOCX) [file pone.0149150.s001.docx]

**Supporting Information (S1 File)**

**A Rapid and Low-Cost PCR Thermal Cycler for Infectious Disease Diagnostics**

Kamfai Chan, Pui-Yan Wong, Peter Yu, Justin Hardick, Kah-Yat Wong, Scott Wilson, Tiffany Wu, Zoe Hui, Charlotte Gaydos, and Season Wong

**1. Detail description on how to construct and program the TTC**

Arms and servo assembly: Two Hitec HS-55 sub-micro servos were mounted onto a ServoCity SPT50 Sub-Micro Pan & Tilt kit ((Servocity, Winfield, KS), and fitted with an arm made of ¼”x ¼” balsa wood that was cut to length. A mounting rack constructed out of 16 ga. floral wire that was shaped to hold four PCR tubes was mounted at the end of the arm. The arm and servos were mounted to the pan and tilt kit with 3M 110 Double-Sided Foam Tape.

Electronics assembly: The servos were controlled with a Pro Micro 5V/16MHz Arduino-compatible microcontroller (Sparkfun, Boulder, CO), powered by 4 AA batteries in a battery pack. A mini pushbutton was utilized to initiate the start of the cycling process. The mini pushbutton and microcontroller were mounted onto a mini breadboard, along with the standard 100 ohm, and 10k ohm resistor necessary to create a pull-down circuit in order for the button to function properly with the microcontroller.

Final assembly: The electronics assembly mounted on the mini breadboard was adhered to the battery pack, and the pan and tilt servo assembly was mounted onto a 305 g can of soup to provide a stable base for operation.

**Arduino Wiring Set up Instructions**

The thermal cycler is patched together using a breadboard. Numbered columns are electrically connected, lettered rows are electrically distinct (**Fig. A**).

**Power Rails – battery assembly to breadboard**

1. Place the positive, red wire (+) from the battery on column 20, hereafter referred to as POWER.

2. Place the negative, black wire (-) from battery onto column 19, hereafter referred to as GROUND.

**Power Arduino microcontroller – Arduino to microcontroller**

1. Place a connecting wire from POWER column to the “5V” terminal of the Arduino microcontroller.

2. Place a connecting wire from GROUND column to a “GND” terminal of the Arduino microcontroller.

**Pan Servo power & control – Servo to breadboard and Arduino microcontroller**

1. Attach the yellow (signal) wire of the “pan” servo to terminal “9” of the Arduino microcontroller.

2. Attach the red wire (+) to the POWER column, and the black wire (-) to the GROUND column of the breadboard.

**Tilt Servo power & control – Servo to breadboard and Arduino microcontroller**

1. Attach yellow (signal) wire of the “tilt” servo to terminal “10” of the Arduino microcontroller.

2. Attach red wire (+) to the POWER column, and the black wire (-) to the GROUND column of the breadboard.

**Button setup – breadboard**

1. Install the pushbutton on row E & F and column 5 & 7 of the breadboard. Ensure that the connected legs are on the same column (**see Fig. B**).

2. Install the 10kΩ resistor (brown-black-orange) onto row D and column 1 & 5 of the breadboard.

3. Attach a connecting wire from row C column 1 to the GROUND column.

4. Install the 100Ω resistor (brown-black-brown) onto row G and column 1 & 5 of the breadboard.

5. Attach a connecting wire from terminal “7” of the Arduino to row H and column 1 of the breadboard.

6. Attach a connecting wire from row G and column 7 of the breadboard to the POWER column of the breadboard.


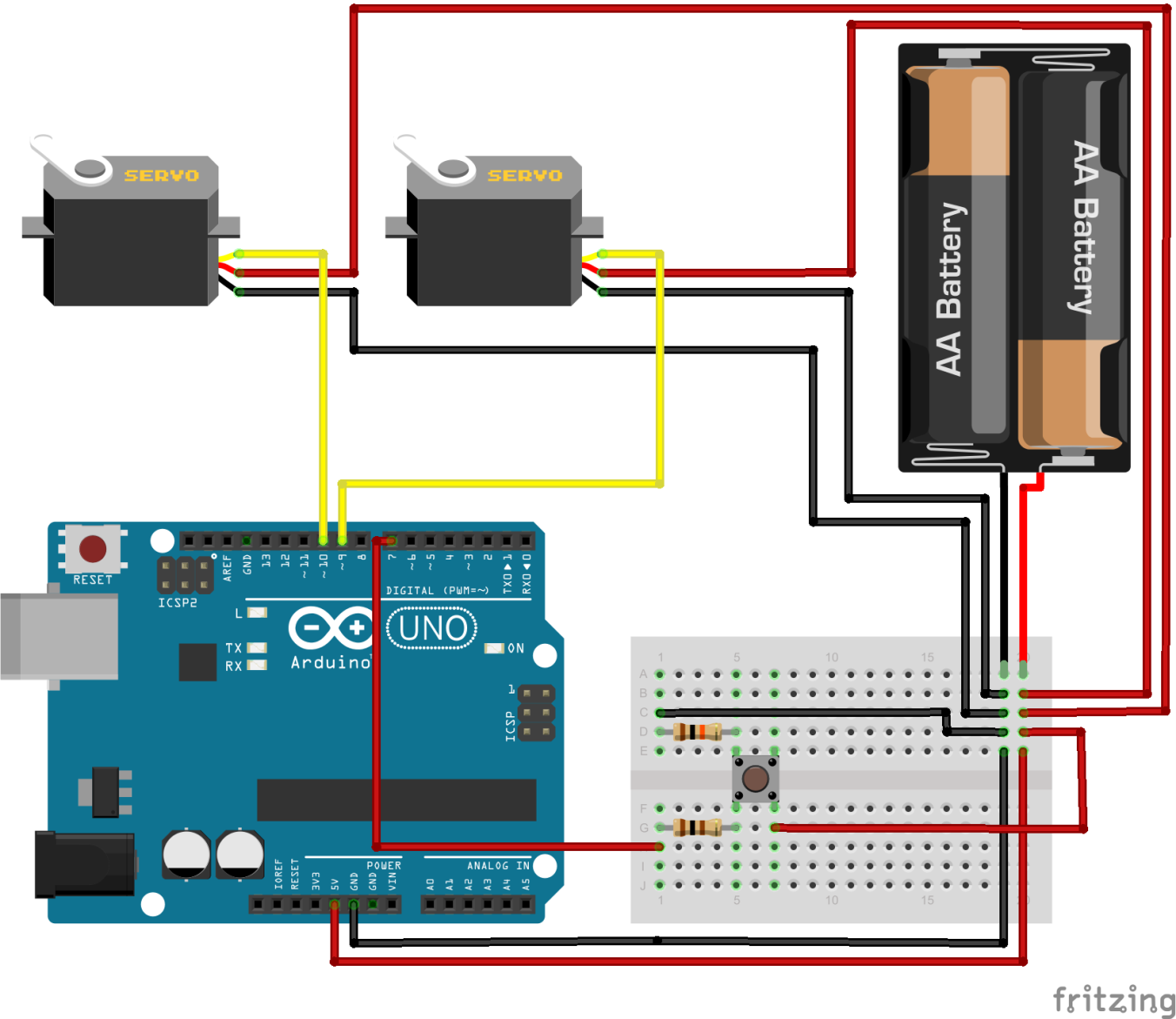


**Fig. A. Overview of TTC assembly wiring with Arduino board.**


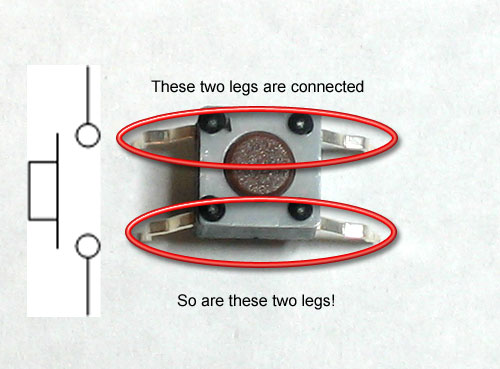


**Fig. B.** **Pushbutton leg configuration.**

**2. Arduino codes for the 4-bath setup to run RT-PCR with room temperature bath**

The following program can be uploaded to the Arduino to control the movement of the pan-and-tilt kit to perform PCR using the TTC. The grey-highlighted sections indicate where users can change the cycle number or the incubation time the tubes spend in each bath. This particular run was 420s/10s/45x(15s/2.5s/30s) and used in the amplification of HIV RNA.

---------------------------------------------------------------------------------------------------------------------

#include <Servo.h> //servo header file

#define LED 13 // the pin for the LED

#define BUTTON 7 // the input pin where the pushbutton is connected

int val = 0; // val will be used to store the state of the input pin

Servo pan; // create servo object to control pan servo

// a maximum of eight servo objects can be created

Servo tilt; // create servo object to control a tilt servo

//NOTE:Here are adjustable values

const int CYCLES = 45; // number of cycles

int startingtiltpoint = 0; // Necessary, but the numerical value does not matter

int startingpanpoint = 0; // Necessary, but the numerical value does not matter

int lowerarmtime = 3; // interval of time (NOT ms) in which the arm lowers into the bath

int raisearmtime = 6; // interval of time (NOT ms) in which the arm raises from the bath

int panarmtime = 4; // interval of time (NOT ms) in which the arm moves from bath to bath

long rthold = 420000; // time (ms) held in the reverse transcription bath (one time only)

long hothold = 10000; // time (ms) held in the denaturation bath (one time only)

long hotdip = 15000; // time (ms) held in the denaturation bath (every cycle)

long roomdip = 2500; // time (ms) held in the room temperature bath (every cycle)

long colddip = 30000; // time (ms) held in the annealing/extension bath (every cycle)

int loweredarmangle = 87; // angle at which the tube are submerged (vertical)

int raisedarmangle = 55; // angle at which the tubes are raised and ready for transport (vertical)

int rtpanangle = 0; // position of the reverse transcription bath

int hotpanangle = 55; // position of the denaturation bath

int roompanangle = 110; // position of the room temperature bath (plastic tubes only)

int coldpanangle = 160; // position of the annealing/extension bath

int tiltanglehold = 45; // Vertical starting position

int pananglehold = 0; // Horizontal starting position

//NOTE: End of adjustable values

void setup() {

pan.attach(9); // attaches the servo on pin 9 to the pan servo object

tilt.attach(10); // attaches the servo on pin 10 to the tilt servo object

pinMode(LED, OUTPUT); // tell Arduino LED is an output

pinMode(BUTTON, INPUT); // and button is an input

}

void loop(){

//Power up

pan.write(pananglehold);

tilt.write(tiltanglehold);

val = digitalRead(BUTTON); // read button value and store it in val

if (val == HIGH) {

//Below is the protocol.

//Here is the start protocol, this portion does not loop, but counts as the first cycle.

for(startingpanpoint = pananglehold; startingpanpoint <= rtpanangle; startingpanpoint += 1) //moves from pananglehold to rtpanagle

{

pan.write(startingpanpoint);

delay(panarmtime); //duration of arm panning

}

for(startingtiltpoint = tiltanglehold; startingtiltpoint <= loweredarmangle; startingtiltpoint += 1) //lowers arm

{

tilt.write(startingtiltpoint);

delay(lowerarmtime); //duration of arm lowering

}

delay(rthold); //holds arm at current position for the duration of rthold

for(startingtiltpoint = loweredarmangle; startingtiltpoint >= raisedarmangle; startingtiltpoint -= 1) //raises arm

{

tilt.write(startingtiltpoint);

delay(raisearmtime); //duration of arm raising

}

for(startingpanpoint = rtpanangle; startingpanpoint <= hotpanangle; startingpanpoint += 1) //moves from rtpanangle to hotpanangle

{

pan.write(startingpanpoint);

delay(panarmtime); //lowers arm within panarmtime duration

}

for(startingtiltpoint = raisedarmangle; startingtiltpoint <= loweredarmangle; startingtiltpoint += 1) //lowers arm

{

tilt.write(startingtiltpoint);

delay(lowerarmtime); //raises arm within lowerarmtime duration

}

delay(hothold); //holds arm at the current position for the hothold duration

delay(hotdip); //holds arm at the current position for the hotdip duration

//End of start protocol

//Here begins the looped protocol, the following instructions will repeat for the remaining number of desired cycles, minus the last cycle (which will follow the ending protocol).

for (int i = 1; i < CYCLES; i++){

for(startingtiltpoint = loweredarmangle; startingtiltpoint >= raisedarmangle; startingtiltpoint -= 1) //raises arm

{

tilt.write(startingtiltpoint);

delay(raisearmtime); //duration of arm raising

}

for(startingpanpoint = hotpanangle; startingpanpoint <= roompanangle; startingpanpoint += 1) //moves from hotpanagle to roompanangle

{

pan.write(startingpanpoint);

delay(panarmtime); //duration of arm panning

}

for(startingtiltpoint = raisedarmangle; startingtiltpoint <= loweredarmangle; startingtiltpoint += 1) //lowers arm

{

tilt.write(startingtiltpoint);

delay(lowerarmtime); //duration of arm lowering

}

delay(roomdip); //holds arm at the current position for the roomdip duration

for(startingtiltpoint = loweredarmangle; startingtiltpoint >= raisedarmangle; startingtiltpoint -= 1) //raises arm

{

tilt.write(startingtiltpoint);

delay(raisearmtime); //duration of arm raising

}

for(startingpanpoint = roompanangle; startingpanpoint <= coldpanangle; startingpanpoint += 1) //moves from roompanangle to coldpanangle

{

pan.write(startingpanpoint);

delay(panarmtime); //duration of arm panning

}

for(startingtiltpoint = raisedarmangle; startingtiltpoint <= loweredarmangle; startingtiltpoint += 1) //lowers arm

{

tilt.write(startingtiltpoint);

delay(lowerarmtime); //duration of arm lowering

}

delay(colddip); //holds arm at the current position for the colddip duration

for(startingtiltpoint = loweredarmangle; startingtiltpoint >= raisedarmangle; startingtiltpoint -= 1) //raises arm

{

tilt.write(startingtiltpoint);

delay(raisearmtime); //duration of arm raising

}

for(startingpanpoint = coldpanangle; startingpanpoint >= hotpanangle; startingpanpoint -= 1) //moves from coldpanangle to hotpanangle

{

pan.write(startingpanpoint);

delay(panarmtime); //duration of arm panning

}

for(startingtiltpoint = raisedarmangle; startingtiltpoint <= loweredarmangle; startingtiltpoint += 1) //lowers arm

{

tilt.write(startingtiltpoint);

delay(lowerarmtime); //duration of arm lowering

}

delay(hotdip); //holds arm at the current position for the hotdip duration

}

//End of the looped cycle protocol

//Ending protocol, this portion does not loop, but counts as the final cycle.

for(startingtiltpoint = loweredarmangle; startingtiltpoint >= raisedarmangle; startingtiltpoint -= 1) //raises arm

{

tilt.write(startingtiltpoint);

delay(raisearmtime); //duration of arm raising

}

for(startingpanpoint = hotpanangle; startingpanpoint <= roompanangle; startingpanpoint += 1) //moves from hotpanagle to roompanangle

{

pan.write(startingpanpoint);

delay(panarmtime); //duration of arm panning

}

for(startingtiltpoint = raisedarmangle; startingtiltpoint <= loweredarmangle; startingtiltpoint += 1) //lowers arm

{

tilt.write(startingtiltpoint);

delay(lowerarmtime); //duration of arm lowering

}

delay(roomdip); //holds arm at the current position for the roomdip duration

for(startingtiltpoint = loweredarmangle; startingtiltpoint >= raisedarmangle; startingtiltpoint -= 1) //raises arm

{

tilt.write(startingtiltpoint);

delay(raisearmtime); //duration of arm raising

}

for(startingpanpoint = roompanangle; startingpanpoint <= coldpanangle; startingpanpoint += 1) //moves from roompanangle to coldpanangle

{

pan.write(startingpanpoint);

delay(panarmtime); //duration of arm panning

}

for(startingtiltpoint = raisedarmangle; startingtiltpoint <= loweredarmangle; startingtiltpoint += 1) //lowers arm

{

tilt.write(startingtiltpoint);

delay(lowerarmtime); //duration of arm lowering

}

delay(colddip); //holds arm at the current position for the colddip duration

for(startingtiltpoint = loweredarmangle; startingtiltpoint >= raisedarmangle; startingtiltpoint -= 1) //raises arm

{

tilt.write(startingtiltpoint);

delay(raisearmtime); //duration of arm raising

}

for(startingpanpoint = coldpanangle; startingpanpoint >= pananglehold; startingpanpoint -= 1) //moves from coldpanangle to pananglehold

{

pan.write(startingpanpoint);

delay(panarmtime); //duration of arm panning

}

//end of protocol.

}

else {

digitalWrite(LED, LOW);

pan.write(pananglehold);

tilt.write(tiltanglehold);

}

}

//Tips: when renaming values, use ctrl+f to replace all to reduce risk of typos and accidental deletions.

**3. Room-temperature bath can speed up PCR when plastic tubes are used**

Temperature probes placed inside of a glass capillary tube (with 10 µL of water) or plastic tube (with 20 µL of water) were used to measure the speed of temperature change when the tubes were moved from a denaturation bath at ~95 °C to an annealing/extension bath at 60 °C versus a room-temperature bath (RTB) at ~22 °C. K-type thermocouples (Model No. SC-TT-K-30-36, Omega Engineering, Stamford, CT) connected to a data logger thermometer (HH147U, Omega Engineering, Stamford, CT) were used.

When moved from the denaturation to the annealing/extension bath, the temperature drop inside the plastic tube was very slow, taking over 13s for the water temperature inside the tube to get near the set annealing/extension temperature (60 °C). However, when the same plastic tube was first placed in the RTB, the temperature of the water inside dropped to slightly over 60 °C in just 3s (we used 2.5 s in the RT-PCR runs to ensure than the temperature does not drop below 60 °C). This means using the servo to move the plastic tube into the RTB bath for a couple of seconds prior to going into the annealing/extension bath will allow the content of the plastic tube reaches the set annealing/extension temperature much faster, even though an extra second is needed to move the tube in and out of the room-temperature bath. Overall, the use of RTB can cut 10 s off each cycle (or near 7 minutes per 40-cycle reaction) when TTC runs plastic tubes.

As for glass capillary tube, the use of RTB had little value. This is because when changing from denaturation to annealing/extension bath at 60 °C, it took less than 3 s for the temperature inside the glass tube to get near the set annealing/extension temperature. Putting the glass tube in the RTB caused the temperature inside to go below 60 °C in just one second and would cause non-specific primer annealing and amplification. Due to the risk of non-specific amplification, we only implemented the use of RTB when using plastic tubes. The following plot shows how fast the temperatures drop, as measured by a temperature probe inside a glass capillary or plastic tube, when the tubes were moved from the denaturation bath to the annealing/extension bath versus a RTB.


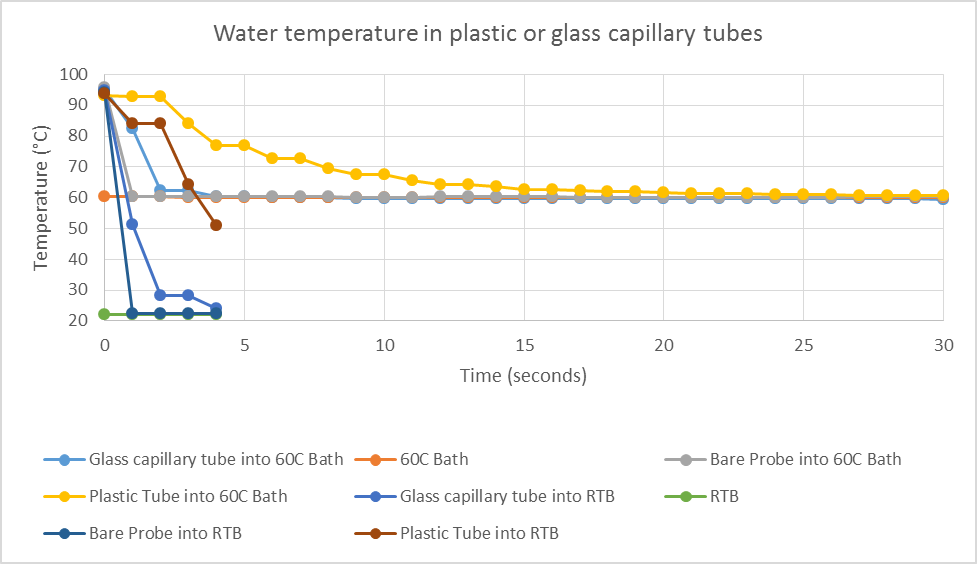


**Fig. C.** **Plot that shows the advantage of using a RTB to shorten the time needed to bring the reagent temperature down from denaturation (~95 °C) to annealing/extension condition (~60 °C)quicker when plastic tubes are used.**
