# Supplementary figures and images for: A Rapid and Low-Cost PCR Thermal Cycler for Infectious Disease Diagnostics
Source: PLoS One. 2016 Feb 12;11(2):e0149150. doi: 10.1371/journal.pone.0149150 (PMC4752298; doi:10.1371/journal.pone.0149150)

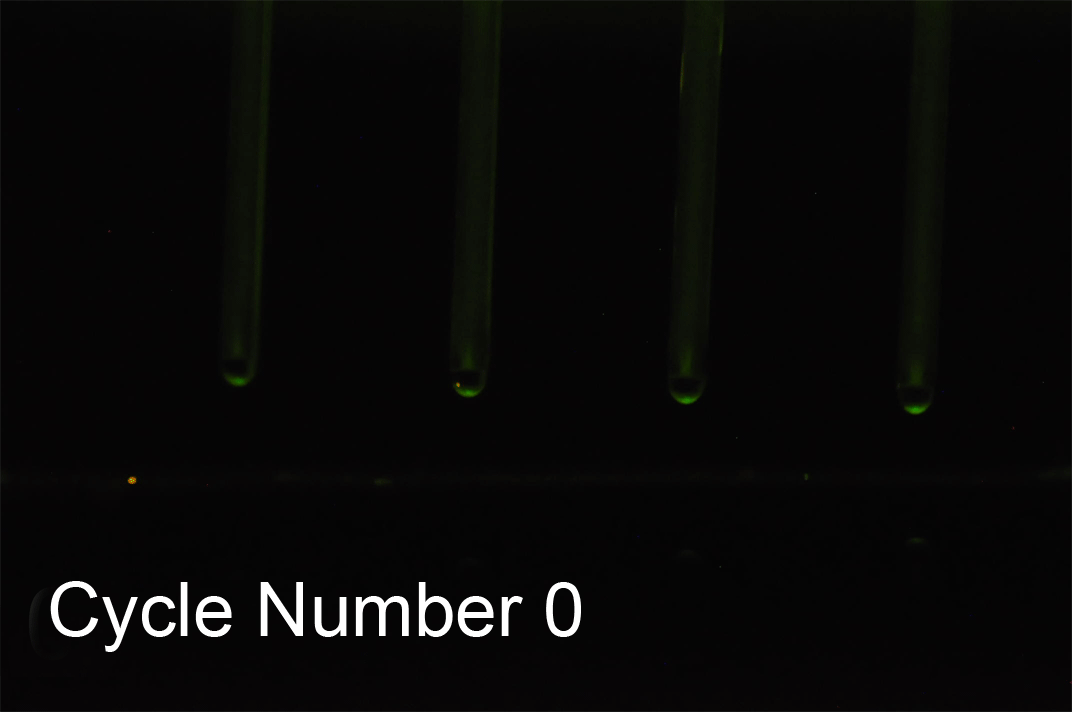

Supplement: S1 Video — The left tube has the highest template concentration (1X), the second tube has 1/10 diluted template, the third tube has 1/1000 diluted template and last tube on the right is the no-template-control sample. As expected, it took the least number of cycles for fluorescence signal in the left tube to rise above the background signal. The fluorescence in no-template control tube remained low throughout the reaction. (GIF) [file pone.0149150.s002.gif]
